# Increased Frequency of Inter-Subtype HIV-1 Recombinants Identified by Near Full-Length Virus Sequencing in Rwandan Acute Transmission Cohorts

**DOI:** 10.3389/fmicb.2021.734929

**Published:** 2021-10-07

**Authors:** Gisele Umviligihozo, Erick Muok, Emmanuel Nyirimihigo Gisa, Rui Xu, Dario Dilernia, Kimberley Herard, Heeyah Song, Qianhong Qin, Jean Bizimana, Paul Farmer, Jonathan Hare, Jill Gilmour, Susan Allen, Etienne Karita, Eric Hunter, Ling Yue

**Affiliations:** ^1^Centre for Family Health Research, Kigali, Rwanda; ^2^Emory Vaccine Center at Yerkes National Primate Research Center, Atlanta, GA, United States; ^3^IAVI, New York, NY, United States; ^4^Faculty of Medicine, Imperial College London, London, United Kingdom; ^5^Department of Pathology and Laboratory Medicine, Emory University, Atlanta, GA, United States

**Keywords:** HIV-1 transmission, genetic bottleneck, HIV subtype, inter-subtype recombination, antiretroviral drug resistance mutations, near full-length genome sequencing

## Abstract

Most studies of HIV-1 transmission have focused on subtypes B and C. In this study, we determined the genomic sequences of the transmitted founder (TF) viruses from acutely infected individuals enrolled between 2005 and 2011 into IAVI protocol C in Rwanda and have compared these isolates to viruses from more recent (2016–2019) acute/early infections in three at risk populations – MSM, high risk women (HRW), and discordant couples (DC). For the Protocol C samples, we utilized near full-length single genome (NFLG) amplification to generate 288 HIV-1 amplicons from 26 acutely infected seroconverters (SC), while for the 21 recent seroconverter samples (13 from HRW, two from DC, and six from MSM), we PCR amplified overlapping half-genomes. Using PacBio SMRT technology combined with the MDPseq workflow, we performed multiplex sequencing to obtain high accuracy sequences for each amplicon. Phylogenetic analyses indicated that the majority of recent transmitted viruses from DC and HRW clustered within those of the earlier Protocol C cohort. However, five of six sequences from the MSM cohort branched together and were greater than 97% identical. Recombination analyses revealed a high frequency (6/26; 23%) of unique inter-subtype recombination in Protocol C with 19% AC and 4% CD recombinant viruses, which contrasted with only 6.5% of recombinants defined by sequencing of the *pol* gene previously. The frequency of recombinants was significantly higher (12/21; 57%) in the more recent isolates, although, the five related viruses from the MSM cohort had identical recombination break points. While major drug resistance mutations were absent from Protocol C viruses, 4/21 of recent isolates exhibited transmitted nevirapine resistance. These results demonstrate the ongoing evolution and increased prevalence of recombinant and drug resistant transmitted viruses in Rwanda and highlight the importance of defining NFLG sequences to fully understand the nature of TF viruses and in particular the prevalence of unique recombinant forms (URFs) in transmission cohorts.

## Introduction

Worldwide 37 million people are living with HIV, two-thirds of these infected individuals are found in Sub-Saharan Africa (UNAIDS, 2020). Even though more than half are receiving ART, a significant fraction of treated patients are not virally suppressed (Hamers et al., 2012; Hauser et al., 2019), and HIV prevention remains a major problem in the fight against HIV. A global effort to design and develop an effective HIV-1 vaccine has been carried out over the last 30years, but one of its major challenges is the enormous diversity of HIV-1. This can be attributed to several factors: the high error rate of the viral reverse transcriptase, since it lacks a proof-reading function; host immune responses that constantly apply selection pressure for less susceptible virus; and the propensity for the virus to undergo recombination (Lukashov and Goudsmit, 1998; Mansky, 1998; Korber et al., 2001; Song et al., 2018). A number of investigators have studied the interplay between HIV-1 and host immunity, and have shown that viral adaptation, particularly to the cellular immune response during the course of infection, can be a major contributor to viral evolution (Moore et al., 2002; Brumme and Walker, 2009; Crawford et al., 2009; Kawashima et al., 2009; Carlson et al., 2012, 2016; Monaco et al., 2016). However, recombination between genetically distinct viruses has the greatest potential to generate diversity (McCutchan et al., 1996; Butler et al., 2007; Lau and Wong, 2013; Giovanetti et al., 2020). HIV-1 has been classified into four phylogenetic groups M, O, N, and P based on nucleic acid sequencing of the viral genomic RNA, with group M being by far the most widespread (Robertson et al., 2000; Desire et al., 2018). The latter is subdivided into nine different subtypes (A–D, F–H, J, K, and the newly identified L), with genetic variation between subtypes ranging from 20 to 35% depending on the genomic regions and the subtypes being compared (Korber et al., 2001; Desire et al., 2018; Yamaguchi et al., 2020). The process of recombination between viruses belonging to different subtypes and the ongoing spread of those recombinants is the basis for the emergence of circulating recombinant forms or CRFs. To date over 102 inter-subtype CRFs have been described (Hemelaar et al., 2020; LANL, 2021). Therefore, to develop a broadly effective prophylactic vaccine, there is a clear need to gain insight into the genotypic and phenotypic features of the viruses from various geographic locations against which a potential vaccine must act.

Rwanda is an East-Central African country bordered by the Democratic Republic of Congo, with a highly diverse HIV-1 population (Rodgers et al., 2017), Burundi, where subtype C is most prevalent (Delatorre and Bello, 2012) and Uganda where subtypes A1 and D predominate, but with a high percentage of unique recombinant forms (URFs; Lee et al., 2017; Grant et al., 2020). Thus, defining the nature of HIV-1 diversity over time in this geographically small, land-locked country will be relevant to ongoing HIV-1 vaccine efforts. Based on a region encompassing the *pol* gene, an earlier subtype analysis of over 90 incident infections enrolled under IAVI Protocol C in Rwanda identified 80% as subtype A1 and only 6.5% as recombinant viruses (Amornkul et al., 2013), while a second study of smaller sample size, where *gag*, *pol*, and *env* genes were sequenced, reported 13.5% recombinant forms in the same cohort (Kemal et al., 2013).

In the current study, we have amplified near full-length single genomes (NFLG) of viruses from the plasma of a total of 26 acutely HIV infected individuals from the Rwandan heterosexual acute infection cohort Protocol C and 21 recently infected individuals from high-risk cohorts. This allowed us to define the sequence of the infecting viruses and compare over two time periods (2005–2012 and 2016–2019) the frequency of inter-subtype recombination across the full genome. In addition, because these two periods define very different availability of anti-retroviral therapies, we were able to compare the prevalence of antiretroviral drug resistance. These data point to an increase in genetic mixing and prevalence of transmitted drug resistance in Rwanda over the last 15years and highlight the importance of NFLG sequencing for assessing diversity in viral populations.

## Materials and Methods

### Ethics Statement

Subjects in this study were enrolled in human subjects protocols approved by the Rwanda National Ethics Committee and Emory University Institutional Review Board. All study subjects have provided written informed consent.

### Study Subjects

In this study, we have studied HIV-1 Early infection subjects from two distinct time periods during last 15years in Kigali Rwanda. During the first period from 2005 to 2011, plasma samples were collected under IAVI Protocol C (Price et al., 2020) from 26/97 (27%) seroconvertors from a heterosexual transmission cohort in Kigali. Individuals enrolled in this cohort were from HIV-1 discordant couples who underwent couples counseling and testing and who were followed, with additional counseling and testing, every 1–3months to reduce the incidence of transmission. HIV infection was identified by p24 ELISA antigen testing or seroconversion. The 26 seroconverters were selected from the original 97 based on the availability of sample during the very early/acute period of HIV infection. During the second period from 2016 to 2019, 21 individuals who were followed in government clinics every 3–6months in virtual cohorts comprised of high-risk women, men having sex with men and discordant couples, were enrolled immediately after seroconversion.

### Viral RNA Extraction and cDNA Synthesis

Viral RNA was extracted from patient’s plasma using the QIAamp RNA mini kit (Qiagen, Valencia, CA). For near-full-length genome amplification, 140μl plasma were used for vRNA isolation, and then the purified vRNA was converted to full-length cDNA with SuperScript III Reverse Transcriptase (Life Technologies) enzyme with a reverse HIV-1 primer that designed at the end of the R region in LTR (Yue et al., 2015). For 5' and 3' half genome amplifications, the amount of plasma sample was calculated according to VL and aliquoted based on 300 copies x # of reaction per each extraction, and then diluted to 140μl with PBS if less than 140μl.

### Near Full-Length Single HIV-1 Genome Amplification

cDNA was serially diluted to yield approximately 30% PCR positive to ensure the majority of amplicons were derived from single virus RNA molecule (Yue et al., 2015). A 9kb PCR fragment extending from the 5' U5 to 3' R region of the genome was generated by using Q5 Hot Start High Fidelity DNA Polymerase (NEB; Deymier et al., 2014). The amplification primers are shown in Table 1 and conditions were as described previously (Deymier et al., 2014).

**Table 1:** cDNA and PCR Primers.

Primer name	Primer sequence (5'-3')	Position in HXB2	Application
OFM19	5'-GCACTCAAGGCAAGCTTTATTGAGGCTTA-3'	9,604–9,632	cDNA synthesis for NFLSGA
1.3'3'plCb	5'-ACTACTTAAAGCACTCAAGGCAAGCTTTATTG-3'	9,611–9,642	cDNA synthesis for NFLSGA
1U5Cc	5'-CCTTGAGTGCTCTAAGTAGTGTGTGCCCGTCTGT-3'	538–571	First round NFLSGA forward primer
1.3'3'plCb	5'-ACTACTTAAAGCACTCAAGGCAAGCTTTATTG-3'	9,611–9,642	First round NFLSGA reverse primer
2U5Cd	5'-AGTAGTGTGTGCCCGTCTGTTGTGTGACTC-3'	552–581	Second round NFLSGA forward primer
2.3'3'plCb	5'-TAGAGCACTCAAGGCAAGCTTTATTGAGGCTTA-3'	9,604–9,636	Second round NFLSGA reverse primer
1half_R1_For	5'-TTTGACTAGCGGAGGCTAGAA-3'	761–781	5'HF first round PCR forward primer
1half_R1_Rev	5'-TTCTATGGAGACYCCATGACCC-3'	5,304–5,283	5'HF cDNA synthesis and first round PCR reverse primer
2half_R1_For	5'-GGGTTTATTACAGGGACAGCAGAG-3'	4,900–4,923	3'HF first round PCR forward primer
OFM19	5'-GCACTCAAGGCAAGCTTTATTGAGGCTTA-3'	9,604–9,632	3'HF cDNA synthesis and first and second round PCR reverse primer
1half_R2_For	5'-TTTGACTAGCGGAGGCTAGAAGGA-3'	761–784	5'HF second round PCR forward primer
1half_R2_Rev	5'-TCCCCTARTGGGATGTGTACTTCTGAAC-3'	5,195–5,222	5'HF second round PCR reverse primer
2half_R2_For	5'-GCAAAACTACTCTGGAAAGGTGAAGGG-3'	4,944–4,970	3'HF second round PCR forward primer

### One-Step RT-PCR Population Half-Genome Amplifications

One-step PCR was conducted using the SuperScript™ III One-Step RT-PCR System with Platinum™ Taq High Fidelity DNA Polymerase (Invitrogen). Master mix I (MMI) contained 25μm reverse first round primer and template vRNA (300 copies per reaction), adjusted to a total volume of 11μl per reaction with H_2_O. The MMI was incubated at 65°C for 5min to melt secondary structures in the RNA then temperature was decreased to 4°C to anneal the 5' or 3' first round reverse primer (Table 1) with the RNA template. Master mix II (MMII) contains 2XReaction buffer, 5' or 3' first round forward primer (25μm; Table 1), SuperScript III/Platinum Taq Mix in a total volume of 39μl per each reaction. The 39μl MMII was added to 11μl MMI at 4°C, and then the entire 50μl reaction was incubated at 55°C for 30min in PCR cycler to synthesize cDNA.

After the cDNA was synthesized, the initial PCR step was 2min at 94°C; followed by 30cycles of 94°C 15s, 52°C 30s, 68°C 6min; and an additional one step of 68°C for 10min for stabilization.

Second round PCR was carried out by using Q5 Hot Start High Fidelity DNA Polymerase (NEB). The sequences and positions of the 5' half second round primers are shown in Table 1. PCR conditions were 98°C 30s as the initial step, followed by 35cycles of 98°C 10s, 64°C 30s and 72°C 4min; plus, an additional step of 72°C 10min prior to keeping the reaction at 4°C.

The sequences and positions of the 3' half second round forward primer and reverse primer OFM19 are shown in Table 1. PCR conditions were 98°C 30s as the initial step, followed by 35cycles at 98°C 10s, 58°C 30s for annealing and 72°C 4min for extension; plus, an additional step of 72°C 10min prior to keeping the reaction at 4°C. The second round PCR resulted in a 4,456bp fragment from the 5'half, and a 4,742bp fragment from the 3'half.

### PacBio DNA Sequencing Library Preparation

Four SMRTbell™ libraries of NFLSGA and four SMRTbell™ libraries of half genome amplicons were built to gain deep sequencing data. The PacBio sequencing method was described previously (Dilernia et al., 2015). In brief, we combined 75 NFLSGA amplicons for each RSII library; 10 patients’ half genome PCR products were collected for each RSII library. The final library DNA concentration was more than 20ng/μl, purity 260/280 ratio was greater than 1.8, and 260/230 ratio was greater than 2.0; total volume was 30μl. SMRT sequencing was performed on a PacBio RSII at the University of Delaware DNA Sequencing & Genotyping Center.

### Sequence Analysis

Data derived from the PacBio RSII was run using the error correction algorithm MDPseq (Dilernia et al., 2015). Defining transmitted founder (TF) viral sequences and phylogenetical analysis were carried out through Geneious v9.1.8 (Biomatters Ltd). Codon-align for each HIV-1 protein was performed by Gene Cutter (LANL). Subtyping and recombinant identification were carried out by Recombinant analysis program (RIP) and jpHMM at GOBICS (LANL).

### GenBank Submission

Near full-length (NFL) sequences were submitted to GenBank. The accession numbers for the 26 Protocol C derived viruses are JX236678.1, JX236677.1, and MT942708-MT942972. Those for the 21 recent seroconverters are MZ642260-MZ642280.

## Results

### Study Volunteers

This project was conducted in partnership with Projet San Francisco/Centre for Family Health Research which was established in Kigali, Rwanda in 1986. Two distinct groups of HIV-1 infected volunteers were studied: Group 1 represented acutely and very-early infected individuals from 2005 to 2011 (IAVI’s Protocol C cohort), while Group 2 represented infected individuals with early HIV infection from 2016 to 2019. For Group 1, HIV discordant couples enrolled in a longitudinal prospective prevention study were provided with counseling, condoms and HIV testing of the seronegative partner during the study (Allen et al., 1992). Couples voluntary counseling and testing (CVCT) in high prevalence areas has been shown to reduce transmission incidence of HIV in cohabiting couples by more than two-thirds (Wall et al., 2019). When infection of the seronegative partner was identified as described in methods, they were enrolled in IAVI Protocol C, an acute infection, long-term follow-up study, and samples were obtained from both partners (Price et al., 2020). Originally, 94 volunteers with incident HIV infection were enrolled into Protocol C from Kigali; in the current study, we analyzed plasma viruses from the 26 seroconverters with the shortest estimated time from the date of infection (median time from EDI=23days) calculated as described in methods; with 16 out of 26 plasma collected less than 30days post-EDI (Table 2). For Group 2, individuals were identified in collaboration with government clinics generally within 3months of their last seronegative visit (median time from EDI=91days; Table 3), so that we could compare the phylogenetics and subtypes of contemporaneous viruses circulating in 2016 through 2019 with those from 2005 to 2011. We analyzed plasma viruses from 21 of these newly infected individuals who included partners of HIV-1 discordant couples (2), female sex workers (FSW; 13), and young MSM (6; Table 3).

**Table 2:** Transmission variants and subtypes in protocol C individuals.

PCID	Coded ID	Sample date	EDI	VL	Transmission variants	Sequence type	Subtype by NFLG	Subtype by pol
175,019	R49M	30-Mar-05	10	3,000,000	Single	TF	A1	A1
175,042	R463F	9-Mar-07	10	152,000,000	Single	TF	A1	A1
175,079	R3103M	19-Sep-08	13	750,001	Single	TF	A1	A1
175,089	R3584M	15-Dec-09	13	223,440	Single	TF	A1C	A1
175,005	R53F	16-Jun-05	14	2,090,040	Single	TF	A1	A1
175,062	R1135M	11-Feb-08	14	1,256,940	Single	TF	A1	A1
175,065	R269M	24-Mar-08	14	31,700,000	Single	Con	A1	A1
175,072	R3137M	28-May-08	14	9,061,540	Single	Con	C	C
175,053	R254F	8-Aug-07	15	1,876,000	Single	TF	A1C	C
175,090	R3469F	15-Apr-10	15	2,920,000	>2	TF(P1)	A1	A1
175,093	R2302M	9-Jul-10	15	3,760,000	Single	TF	A1	A1
175,094	R3843F	14-Oct-10	16	4,400,000	Single	TF	A1	A1
175,059	R977F	14-Dec-07	17	7,290,000	Single	TF	A1	A1
175,038	R880F	12-Jan-07	21	730,000	Single	TF	A1	A1
175,097	R3894M	18-Mar-11	23	6,934,997	Single	TF	CD	CD
175,092	R3671F	8-Jun-10	25	3,940,000	Single	NA	A1	A1
175,074	R873F	24-Jul-08	37	219,920	2	TF(P1)	A1	A1
175,071	R1077F	12-Jun-08	41	4,702,444	3	TF(P2)	A1	A1
175,008	R50M	25-May-05	45	117,608	>2	NA	C	C
175,020	R57F	12-Oct-05	46	134,472	>2	NA	A1	A1
175,014	R59M	21-Oct-05	46	806,290	Single	TF	A1	A1
175,017	R44M	3-May-05	49	123,560	Single	TF	A1C	A1
175,011	R63M	14-Dec-05	50	184,270	Single	Con	A1C	A1C
175,012	R40F	31-Mar-05	53	1,398,004	Single	TF	A1C	A1
175,027	R72M	10-Aug-06	67	425,000	>2	NA	A1	A1
175,010	R65M	27-Jan-06	73	148,220	Single	TF	A1	A1

PCID, protocol C identification number of the participant; Coded ID, coded identification number used in two previous publications (Haaland et al., 2009; Yue et al., 2015); Sample date, date of sample collection; EDI, time since estimated date of infection; VL, viral load of sample; Transmission variants, number of genetic variants transmitted from partner; Sequence type, sequence defined as TF: transmitted founder virus sequence amplicon identical to consensus identified in NFLG amplicons; (P1), (P2): TF sequence identified in subpopulation 1 or 2 of multiple variants, Con: consensus sequence of NFLG amplicons: Subtype by NFLG: subtype as deduced from the NFLG sequence, Subtype by pol: Subtype defined previously from pol gene sequencing.

**Table 3:** Summary of recent infection samples and derived near full-length sequences.

Coded ID	Sample date	EDI	VL	Subtype	Risk group^*^
BUS71F	10-July-19	91.5	328,000	A1	FSW
GIT84F	1-August-19	131	102,000	A1	FSW
GWE47F	10-October-18	111.5	150,000	A1	FSW
GWE68F	25-June-19	216	120,000	A1	FSW
KAG34F	10-November-17	88	191,000	A1/C/D	FSW
KIN18F	17-March-17	46	113,000	A1	FSW
MAS1F	18-August-16	44.5	553,000	A1	FSW
MAS21F	30-March-17	164.5	856,000	A1	DC
MAS6M	22-November-16	174.5	202,000	A1/C	DC
MAS7F	23-November-16	46	47,200	A1/C	FSW
MAT81F	24-July-19	181	292,000	A1/C/D	FSW
NGA76F	17-July-19	90	141,000	A1/C	FSW
NGA77F	17-July-19	90	473,000	A1/C	FSW
PSF24M	2-June-17	96	306,000	A1/C	MSM
PSF33M	20-October-17	194	160,000	A1/C	MSM
PSF36F	4-January-18	94	702,000	A1/C	FSW
PSF38M	17-September-18	174	145,000	A1/C	MSM
PSF39M	17-February-18	72	100,000	A1/C	MSM
PSF3M	21-October-16	51	86,500	A1/C	MSM
PSF80M	19-July-19	17	683,000	C	MSM
REM29F	24-August-17	91	148,000	A1	FSW

Coded ID, coded identification number of the participant; sample date, date of sample collection; EDI, time since estimated date of infection; VL, viral load of sample; subtype, subtype defined by near full-length genomic sequence; risk group, risk group of the individual, FSW: female sex worker; DC, HIV discordant couple; and MSM, men having sex with men.

### Severity of Genetic Bottleneck During HIV Transmission in the Protocol C Heterosexual Acute Transmission Cohort

**Figure 1:**
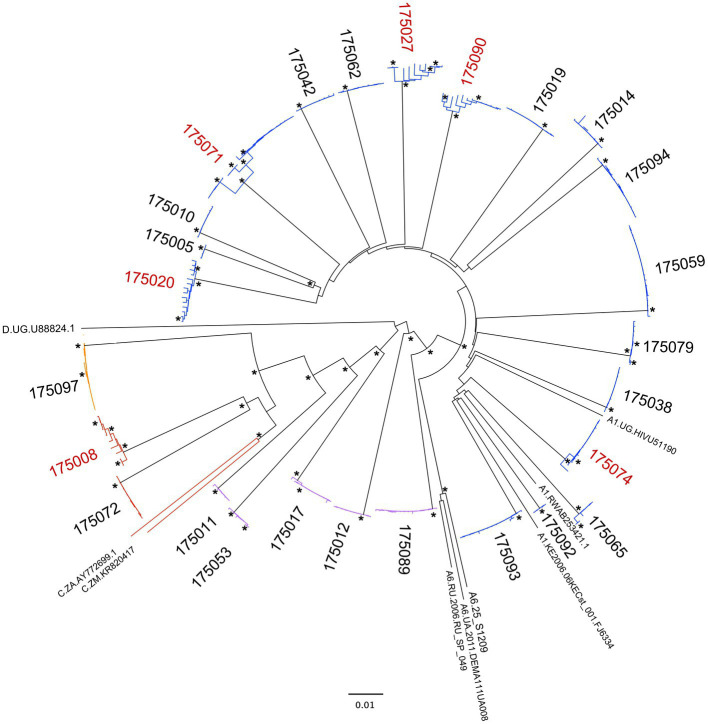
Phylogenetic analysis of virus sequences from Protocol C acute infections. Neighbor-joining tree representing the near full-length genome (NFLG) sequences of viruses from 26 acutely infected recipients with reference subtypes A1, A6, C, and D. Subtype and recombinants are indicated with specific colors (Subtype A, Blue; Subtype C, Red; Recombinant AC, Purple; Recombinant CD, Orange). The IDs of subjects infected with more than one viral variant from their partner are highlighted in red. Nodes with bootstrap values>0.9 are denoted with an asterisk.

**Figure 2:**
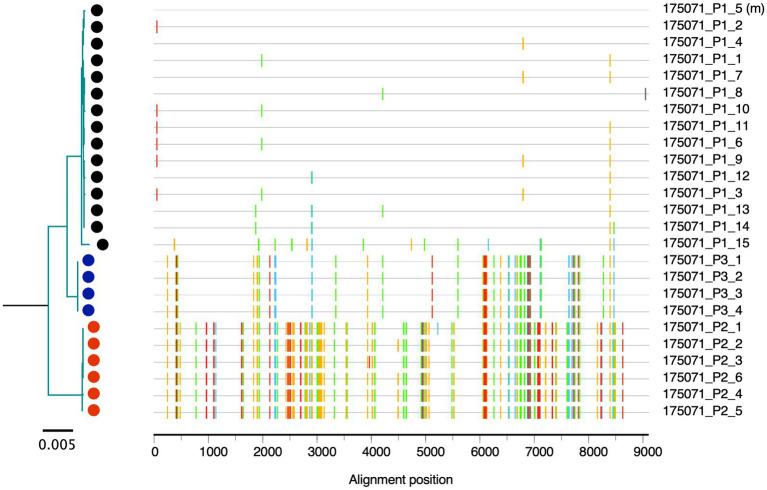
Multi-variants transmission. The phylogenetic tree and aligned sequence highlighter analysis show 175,071 contains three viral populations at the first visit time-point (EDI=41). Tick marks indicate nucleotide change from the master at same position according to the alignment. The color codes are as follows: A: green, T: red, G: yellow, C: blue, and gaps: gray.

In sub-Saharan Africa, heterosexual transmission remains the predominant mode of infection by HIV-1, and accounts for approximately 75% of newly infected cases worldwide (Monaco et al., 2017). We performed near full-length, single genome amplification (NFLSGA ~9,000bp) on HIV-1 from the 26 acute plasma samples using a high-fidelity nested PCR approach described previously (Rousseau et al., 2006; Deymier et al., 2014; Yue et al., 2015; Kinloch et al., 2019). A total of 288 PCR amplicons from viral RNA were sequenced with an average of 11 NFLSGAs per individual using a multiplexed, highly accurate, DNA sequencing approach based on the PacBio Sequencing platform combined with the MDPseq work flow we have described previously (Dilernia et al., 2015). Phylogenetic analysis of the near full-length genome sequences showed that in 20 out of 26 Rwandan seroconverters a single virus variant established infection, with sequences for each individual clustering in a very homogeneous rake (Figure 1). In contrast, in six of 26 individuals, infection was initiated by more than one virus variant (Figure 1); as an example, for the acutely infected individual 175,071 three clearly distinct sequence branches were observed in the phylogenetic tree and three different populations were shown by the highlighter analysis (Figure 2).

### Inter-Subtype Recombinants Recognized by Near Full-Length Sequencing

**Figure 3:**
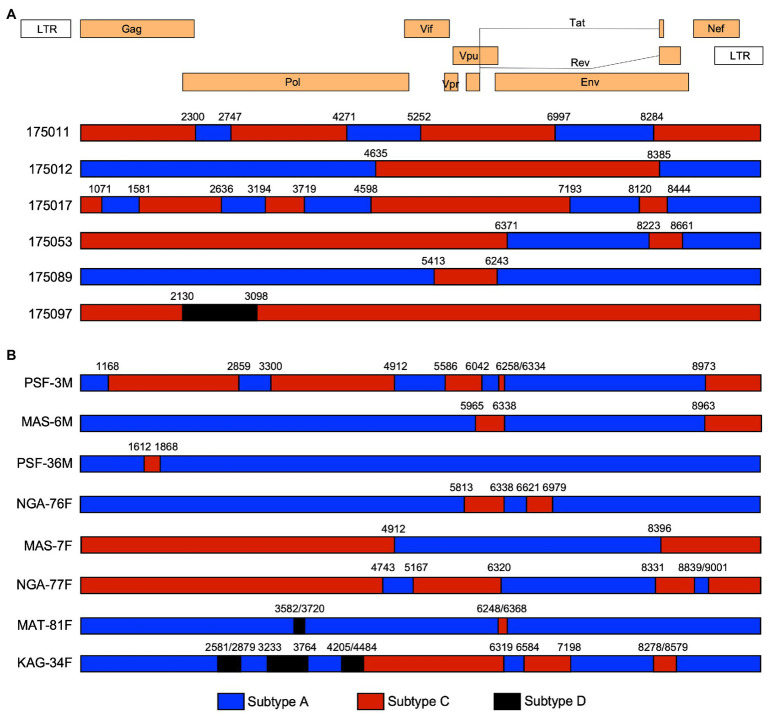
Recombination analysis of Protocol C and recent infections. The positions of recombination cross-overs and the subtype origin of genomic regions are shown based on the jphMM analytical tool. Nucleotide positions refer to the equivlent positions on the HXB-2 HIV-1 genome. **(A)** The six unique recombinant forms (URFs) from the Protocol C acute infection cohort. **(B)** The recombination structure of recent Rwandan seroconvertor viruses. PSF-3M illustrates the identical recombinant structure found in all five early isolates from MSM (PSF-3M, -24M, -33M, -38M, and -39M). Subtype sequences are denoted by the same colors as in Figure 1.

Worldwide HIV-1 Group M is the major source of the global pandemic. In this group there are 9 subtypes, over 100 CRF, and many URF (Hemelaar et al., 2020; LANL, 2021). Globally, the proportions of recombinants has increased over time, reaching almost 23% of all infections in the period 2010 to 2015 (Hemelaar et al., 2020), and URF infections occur frequently in the regions and countries where more than one subtype circulate (Tebit and Arts, 2011). Based on previous subtyping of HIV-1 infection for Protocol C volunteers which employed *pol* gene sequences (Fabiani et al., 1998; Amornkul et al., 2013), we expected that the 26 viruses would comprise approximately 21 Subtype A1 (81%); three subtype C (11%); and two Recombinant (8%, 1 A1/C and 1 C/D). In contrast, utilizing the 9kb near full-length genome sequences and the programs RIP and jpHMM^1^ to detect recombinants, we observed a 3-fold higher percentage of recombinant viruses, with six (5A1/C and 1C/D) recombinants (23%) in addition to 18 subtype A1 (69%), and two subtype C (8%) infections (Figures 1, 3A and Table 2). Each of the recombinant genomes exhibited unique recombination breakpoints (URFs), with two viruses 175,011 and 175,017 resulting from multiple crossovers between their subtype A1 and C progenitors (Figure 3A). A majority of the A1/C recombinants retained a portion of the Env gene and/or Nef from their subtype A parent, but no single region was conserved in all five of these A1/C recombinants. Of these the five A1/C URFs and 1 C/D URF were single virus transmission cases: while one of two subtype C and five of 18 subtype A1 involved multi-variant transmission (Figure 1).

### Near Full-Length Sequencing of Plasma Virus in Recent Seroconverters

**Figure 4:**
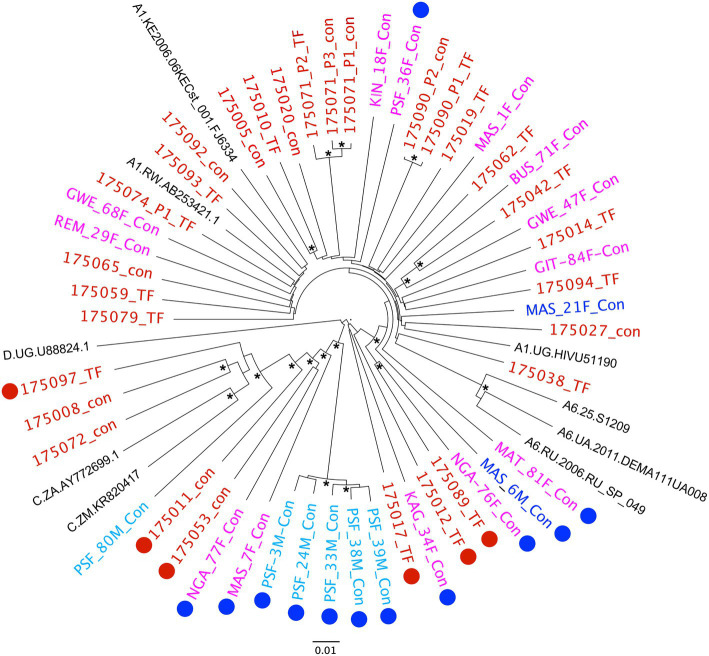
Phylogenetic analysis of virus sequences from recent infections in the context of Protocol C acute infections. Neighbor-joining tree representing NFLG sequences of viruses from 21 recent infections and 26 acute Protocol C recipients. Origin of the viruses is denoted by color – Red, Protocol C; Blue, Discordant Couple; Magenta, FSW; Cyan, MSM. Recombinant viruses are highlighted by appended Red (Protocol C) and Blue (Recent Infection) circles. Nodes with bootstrap values>0.9 are denoted with an asterisk.

Near full-length (9kb) single genome amplification is very inefficient, expensive and time consuming, therefore, for recent samples, we opted to utilize the more efficient population PCR amplification of 5' and 3' half-genome regions, with amplicons that overlapped by ~250 nucleotides. The population amplicons were then sequenced using next-generation PacBio single molecule real time sequencing and individual reads were analyzed using the MDPseq workflow. This allowed us to determine the consensus sequence for the early virus population and breakpoints in those determined to be recombinants. A phylogenetic analysis of the 21 recent seroconversion viruses in the context of the 26 viruses from Protocol C (Red; Figure 4) shows that overall, the two viruses from discordant couple transmissions (Blue) and a majority of the viruses from newly infected FSWs (Magenta) clustered within the diversity of the older viral isolates. In contrast five of the six newly infecting viruses from the cohort of young MSM (Cyan) clustered together on the phylogenetic tree with the sequences exhibiting very limited diversity (median 97.5% identity). This would be consistent with a recent transmission network within a risk group that otherwise has demonstrated low sero-incidence (Karita et al., in preparation).

An analysis for recombination using the RIP and jpHMM tools^2^ revealed that 12 of the 21 recent seroconversions were initiated by unique A1/C and A1/C/D recombinant forms (Figure 4, denoted by blue circles). These represent, therefore, 57% of the samples analyzed, a significantly higher frequency than we observed in the Protocol C samples (23%; *p*=0.033). Even if we define the five closely related viruses from MSM as a single recombinant, the frequency (47.1%) while no longer significant (*p*=0.182) remains double that of the earlier samples.

Interestingly, while each of the recent recombinants exhibited unique numbers and positions of crossovers (Figure 3B), a region of subtype A1 Env, extending from just before an 18-residue amphipathic alpha-helical region located on the outer domain of gp120, known as the alpha 2 helix, to the membrane-spanning domain of gp41, was conserved throughout. A similar, albeit shorter region that terminated in the C-terminal heptad repeat of gp41, was present in four of the five unique A1/C recombinants identified in the Protocol C samples (Figure 3A).

### Identification of Antiretroviral Drug Resistance Mutations

Finally, in order to determine whether, in the context of increased availability of ART, transmitted drug resistance was increasing, sequences encompassing the protease, reverse transcriptase, and integrase were extracted from all 47 near full-length genomes and submitted to the Stanford HIVdb analysis program, and any major drug resistance mutations (DRMs) documented. None of the Protocol C viral sequences analyzed for this study encoded any major DRMs. In contrast, four of the 21 recent isolates encoded DRMs in the reverse transcriptase. MAS-21F, MAS-1F, and PSF-80M encoded the K103N mutation that confers high level resistance to the non-nucleoside inhibitors nevirapine (NVP) and efavirenz (EFV). NGA-77F encoded K103S, which confers high level resistance to NVP and intermediate resistance to EFV, and E138A, which confers low level resistance to etravirine and rilpivirine.

## Discussion

Here, we report on the NFLG sequences of HIV from 47 acute and early infected Rwandans. This more than quadruples the number of near full-length sequences previously reported for this East African country (Lamers et al., 2016). For the IAVI Protocol C samples, we amplified more than 10 NFLG single-genome amplicons per individual, a total of 288, from acute infections that allowed us to define a TF virus sequence in a majority of cases. This was facilitated by the use of PacBio single molecule real-time (SMRT) long-read sequencing combined with the MDPseq workflow, to define accurate sequences (Dilernia et al., 2015), We utilized the same workflow for the more efficient, overlapping half-genome population amplicons generated from recent infections. The long-read technology allowed the population PCR to be deconvoluted to yield the individual sequences of the corresponding amplicons in order to generate a consensus sequence of the infecting virus.

We have previously reported, based on sequencing of the V1-V4 region of Env that in a majority of transmission events in both a Zambian discordant couple cohort and the Rwandan Protocol C cohort infection was established by a single genetic variant from the transmitting partner (Derdeyn et al., 2004; Haaland et al., 2009). In general, multiple variant infections represented only 10–15% of the transmission pairs examined (Haaland et al., 2009). In the current study, we observed a somewhat higher frequency (23%) of infections initiated by more than one partner-derived virus variant, similar to that reported in a South African women cohort (Abrahams et al., 2009) and a predominantly MSM cohort (Keele et al., 2008). We have reported previously that evidence of genital inflammation and ulcers can lower the barrier to transmission and increase multi-variant transmission (Haaland et al., 2009; Carlson et al., 2014), and a recent study in a Kenyan MSM cohort, where sexually transmitted infections were common reported 39% (15 out of 38) of the participants were infected with multiple founder viruses (Macharia et al., 2020). In the heterosexual protocol C cohort studied here we observed evidence of genital inflammation or ulcers in just six individuals in the 6months prior to HIV-1 infection and these individuals were equally distributed between the single and multiple variant infections groups (*p*=0.67; Chi-square with Yates correction).

Inter-subtype recombination represents a significant challenge to HIV-1 vaccine design since it provides the virus with a mechanism to rapidly diversify following co-infection of an individual with two different subtype viruses (Robertson et al., 1995). This gives rise to unique recombinant forms that can be prevalent in regions where more than one subtype circulates. An example of this is Uganda, Rwanda’s neighbor, where subtypes A and D cocirculate, and recent studies have identified a frequency of recombinants between the two subtypes as high as 49% (Lee et al., 2017; Grant et al., 2020). In contrast, a previous analysis of viral subtypes from 30 women in Rwanda in 2013 reported 80% subtype A1, 3% subtype C and D, and 13% AC or AD recombinant forms based on sequencing of *gag*, *pol*, and *env* (Kemal et al., 2013). Similarly, an earlier subtype analysis of 92 IAVI Protocol C incident infections in Rwanda, which was based on a region encompassing the *pol* gene, defined 80% as subtype A1 and only 6.5% as recombinant viruses (Amornkul et al., 2013). By contrast, using NFLG sequences, we identified 23% (6/26) recombinants, a much higher frequency than even the 8% (2/26) previously defined for these same individuals through *pol* sequencing. This highlights the importance of NFLG sequencing to fully understand the complexity of virus populations circulating in multi-subtype countries. Indeed, sequencing of 21 more recent (2016–2019) incident infection viruses from different high-risk groups suggests that recombinant viruses are increasing in frequency, since we observed that 57% of these recent infections were A1/C, or A1/C/D recombinants. With the exception of the five viruses that appeared to represent a recent viral transmission network, the recombinants in these recent infections and those in Protocol C resulted from a series of unique recombination events. In contrast, five of the six viruses from the MSM cohort were highly related and had identical recombination patterns. Of interest, one common recombination breakpoint (4912) was shared by this MSM group (represented by PSF-3M, Figure 3B) and its nearest neighbor MAS-7F (Figure 4) raising the possibility that the former evolved from the latter following further recombination events. Although, the risk groups in Protocol C and the recent infection cohorts are different, the majority of infections in the latter represented heterosexual transmission in high-risk women and discordant couple partners (15/21), where recombinant viruses remained prevalent (47%). Nevertheless, this difference in risk groups should be considered a potential weakness of the comparison.

Although, a majority of the recombinant viruses from the recent seroconverters exhibited unique recombination break points, they all retained a common region of Env that was derived from a subtype A1 parent. This region extended minimally in KAG-34F from the last few residues of the third variable (V3) loop of gp120, just before the hydrophobic alpha 2 helix, to the beginning of the membrane spanning domain of gp41. We have shown previously, that, in subtype C viruses, the alpha 2 helix is under positive selection pressure and that variations in this region are in part linked to early neutralizing antibody escape (Rong et al., 2007). The conserved region also spans the fifth conserved domain (C5) of gp120 and the ectodomain of gp41, both critical for gp120 and gp41 interactions and trimer stability (Binley et al., 2000; Julien et al., 2013). Thus it is possible that this region from subtype A1 provides a fitness advantage to the recombinants.

We have recently reported on the presence of DRMs in the transmitted virus of newly infected partners of Rwandan couples, where the transmitting partner was on ART but carried drug resistant virus (Woodson et al., 2018). It was of interest, therefore, to compare the prevalence and nature of DRMs in the Protocol C cohort, from a time when antiretroviral drugs were not widely available, to those in more recently infected individuals, when treatment with the standard first-line combination of Tenofovir (TDF), Lamivudine (3TC), and EFV following diagnosis was routine. Although none of the 26 acutely infected individuals from Protocol C encoded DRMs, a previous study of 78 Rwandans from the same cohort did identify five individuals with NNRTI mutations (three with K103N, two with L100I) and one with the protease inhibitor mutation, M46L (Price et al., 2011). Our finding that four of the 21 recent infection viruses encoded K103N/S, which confers resistance to NVP and EFV, indicates that this mutation is becoming more prevalent (*p*=0.035) within the population. Moreover, since all of these samples were collected prior to the initiation of ART, for the three females whose virus encoded this mutation, the resistance mutations must have been present in the virus of their male partners.

Overall, we demonstrate here, through NFLG sequence analysis that recombinant viruses are more prevalent in Rwanda than previously reported, and that the frequency of both recombinants and NNRTI DRMs appear to have increased between two sampling periods. It will be critical therefore to continue to monitor the nature of circulating viruses to ensure the validity of ongoing vaccine development efforts.

## Data Availability Statement

NFL sequences were submitted to GenBank. The accession numbers for the 26 Protocol C derived viruses are JX236678.1, JX236677.1, MT942708-MT942972. Those for the 21 recent seroconverters are MZ642260-MZ642280.

## Ethics Statement

The studies involving human participants were reviewed and approved by Rwanda National Ethics Committee and Emory University Institutional Review Board. The patients/participants provided their written informed consent to participate in this study.

## Author Contributions

LY, EH, JG, SA, and EK conceived and designed the experiments. GU, EM, EN, RX, DD, KH, HS, and QQ performed the experiments. LY, GU, EM, and EN analyzed the data. LY, EH, PF, JH, JB, EK, JG, and SA contributed reagents, materials, and analysis. EH and LY wrote the paper. All authors contributed to the article and approved the submitted version.

## Funding

This work is made possible in part by NIH grant R01AI051231 and by IAVI contract # 06-646-9933 through the support of the American People through the United States President’s Emergency Plan for AIDS Relief (PEPFAR) through the United States Agency for International Development (USAID). The contents of this manuscript are the sole responsibility of the authors and do not necessarily reflect the views of PEPFAR, USAID, or the United States Government. This work was also supported, in part, by the Virology Core at the Emory Center for AIDS Research by performing viral load determinations (grant P30 AI050409); the Yerkes National Primate Research Center base grant through the Office of Research Infrastructure Programs /OD P51OD11132. EH is a Georgia Eminent Scholar.

## Conflict of Interest

The authors declare that the research was conducted in the absence of any commercial or financial relationships that could be construed as a potential conflict of interest.

## Publisher’s Note

All claims expressed in this article are solely those of the authors and do not necessarily represent those of their affiliated organizations, or those of the publisher, the editors and the reviewers. Any product that may be evaluated in this article, or claim that may be made by its manufacturer, is not guaranteed or endorsed by the publisher.

## Footnotes

^1^ www.hiv.lanl.gov

^2^ https://www.hiv.lanl.gov/content/sequence/HIV/HIVTools.html
